# Supplementary figures and images for: Shallow whole genome sequencing for robust copy number profiling of formalin-fixed paraffin-embedded breast cancers
Source: Exp Mol Pathol. 2018 Jun;104(3):161–9. doi: 10.1016/j.yexmp.2018.03.006 (PMC5993858; doi:10.1016/j.yexmp.2018.03.006)

Supplementary Figure 1a

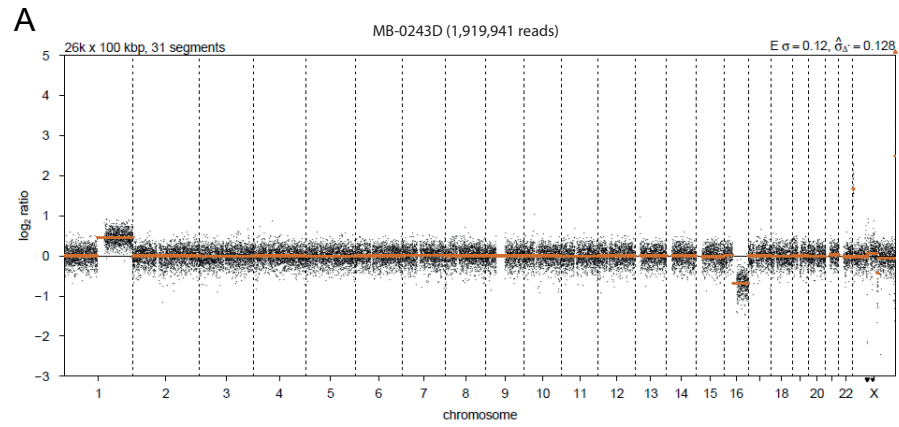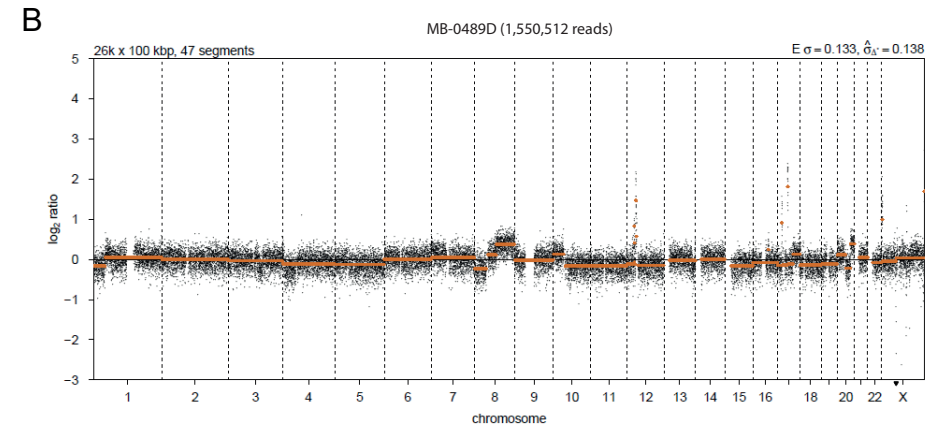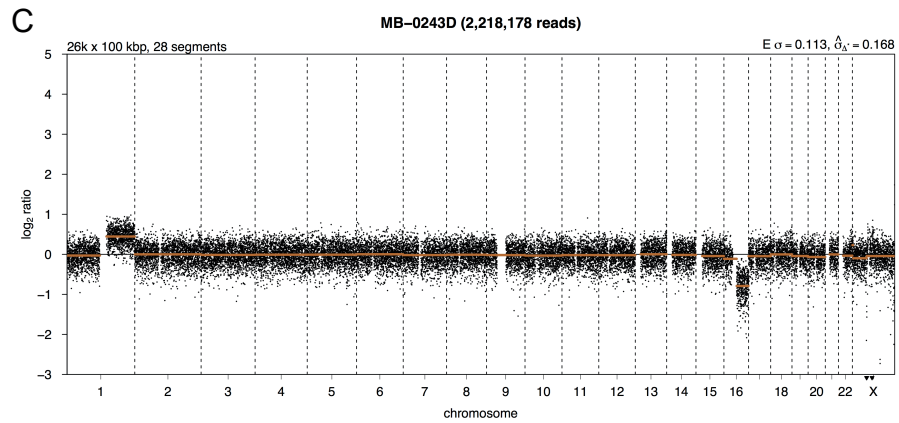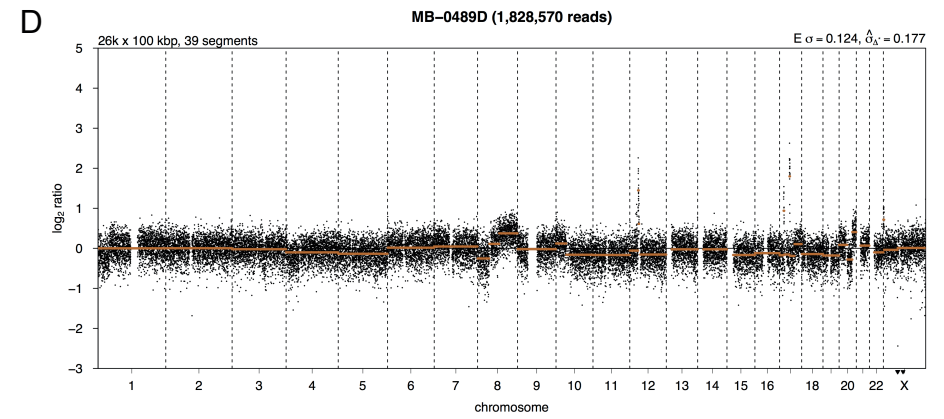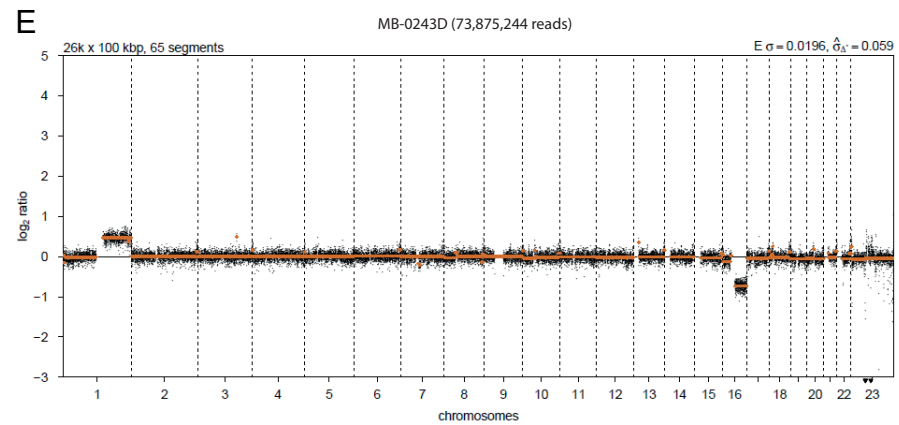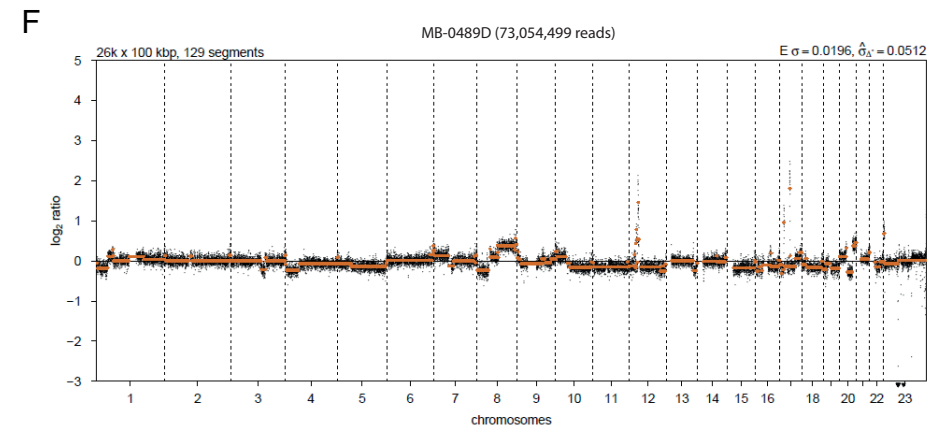

Supplement: Supplementary Fig. 1 — a–b Four separate libraries made with different kits - QDNASEQ copy number plots from four samples made with Rubicon Genomics Thruplex (A, B) or Illumina TruSEQ (C–F). We downsampled the Illumina TruSEQ (C, D) to achieve a more comparable results to the Rubicon Genomics Thruplex (A, B). The actual sequencing depth for Illumina TruSEQ is presented in plot E-F. [file mmc1.pdf]

# Supplementary Figure 1b

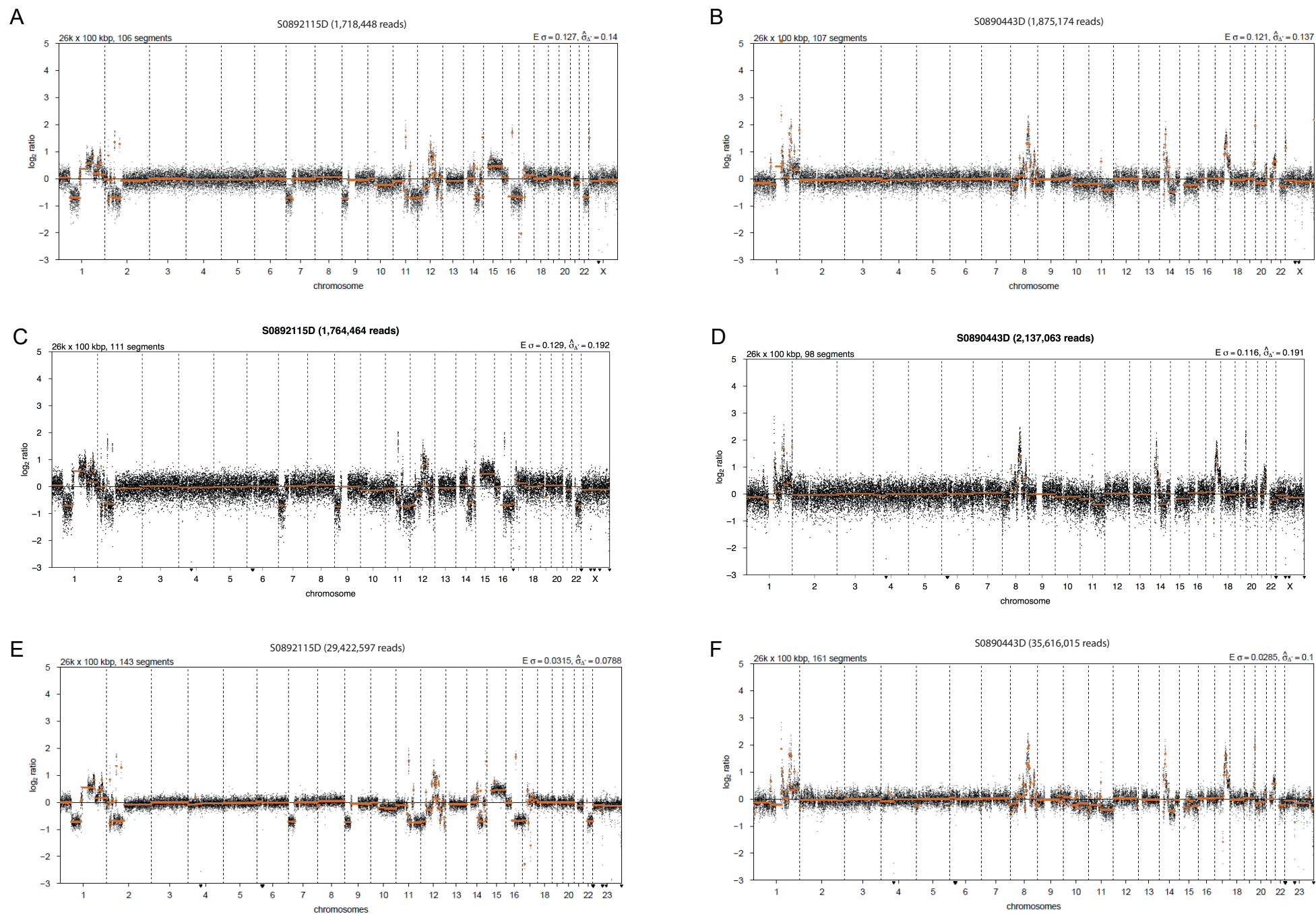

Supplement: Supplementary Fig. 1 — a–b Four separate libraries made with different kits - QDNASEQ copy number plots from four samples made with Rubicon Genomics Thruplex (A, B) or Illumina TruSEQ (C–F). We downsampled the Illumina TruSEQ (C, D) to achieve a more comparable results to the Rubicon Genomics Thruplex (A, B). The actual sequencing depth for Illumina TruSEQ is presented in plot E-F. [file mmc2.pdf]

Supplementary Figure 2a

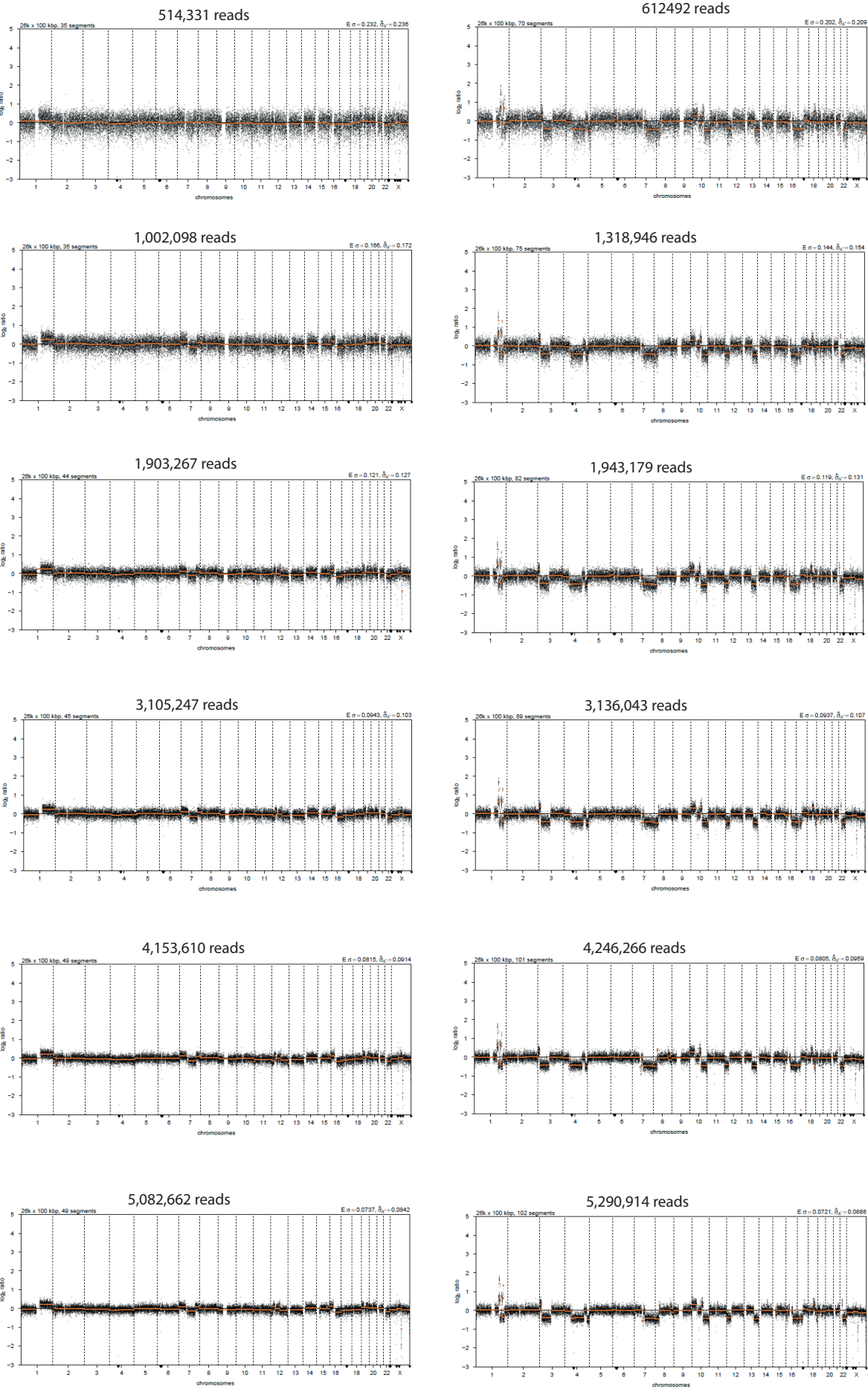

Supplement: Supplementary Fig. 2a — Increasing sequencing depth improves the resolution of the copy number plots - QDNASEQ copy number plots from two samples made with Rubicon Genomics Thruplex with increasing read depth. Sample on the left has a ΔCt of 4.79 and on the right has a ΔCt of 4.31. [file mmc3.pdf]

Supplementary Figure 2b

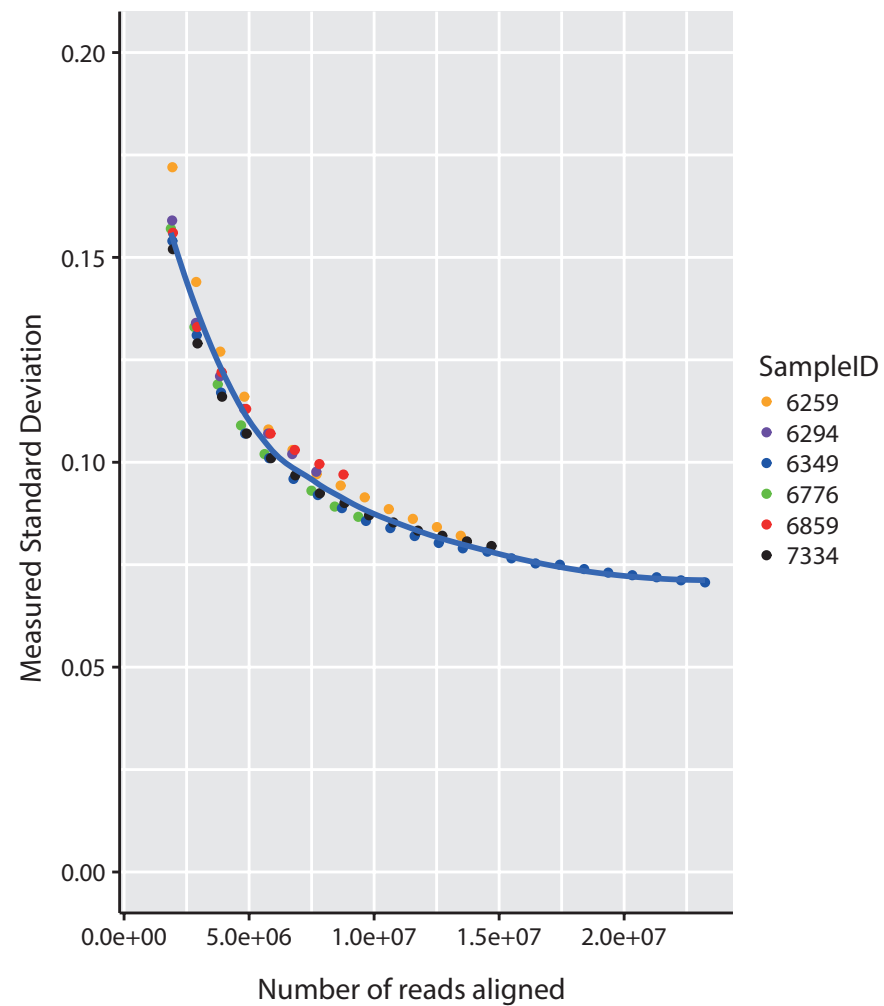

Supplement: Supplementary Fig. 2b — Increasing sequencing depth improves the resolution of the copy number plots – For six libraries, the number of reads were down-sampled stepwise at 1 × 10^6^ reads and plotted against the measured standard deviation (from QDNASEQ plots). [file mmc4.pdf]

Supplementary Figure 3

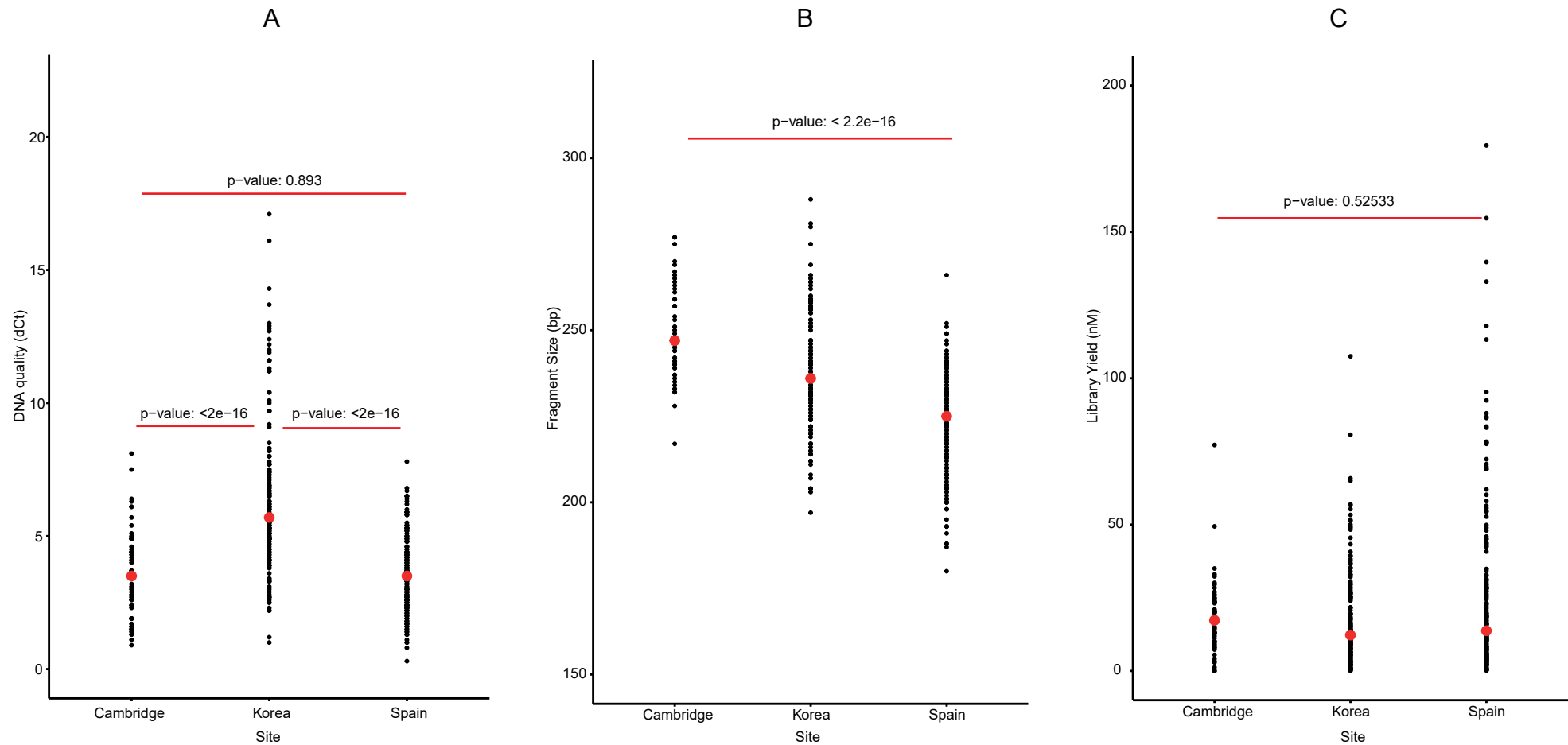

Supplement: Supplementary Fig. 3 — Comparing the input DNA and libraries generated from different sites. Dot plots represent the range (minimum-maximum) observed values for each category and the red dot (•) represents the median. Lines represent comparison between sites with associated statistical p-value. A. The quality of input DNA inferred by ΔCt.B. Fragment sizes of the libraries in base pair. C. The library yield in nanomoles. [file mmc5.pdf]

Supplementary Figure 4

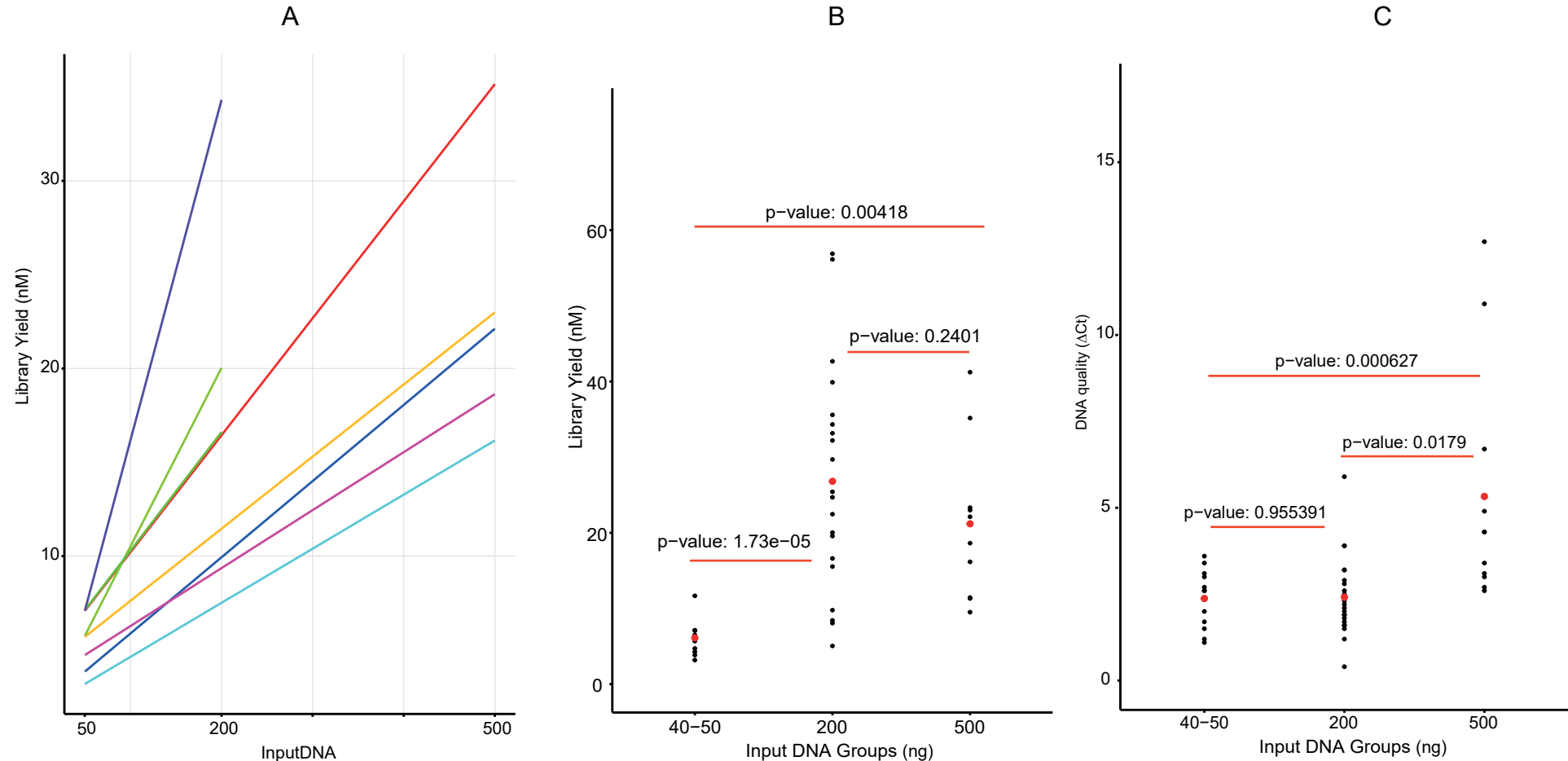

Supplement: Supplementary Fig. 4 — Library yield and its association with amount of input DNA using the Illumina TruSEQ kit. A. For eight samples, libraries were generated with 50 ng and either 200 or 500 ng of input DNA. Each coloured line represents paired libraries drawn connecting the lower and higher input DNA for each sample. B. Dot plots represent the range of library yield when generated with different amount of input DNA and the red dot (•) represent median. Red line represents statistical test between the groups spanning the line with p-value. C. Dot plot represent range of input DNA quality inferred by ΔCt for the samples from the different groups of input DNA and the red dot (•) represent median. Red line represents statistical test between the groups spanning the line with p-value. [file mmc6.pdf]
