# Supplementary material for: Shallow whole genome sequencing for robust copy number profiling of formalin-fixed paraffin-embedded breast cancers
Source: Exp Mol Pathol. 2018 Jun;104(3):161–9. doi: 10.1016/j.yexmp.2018.03.006 (PMC5993858; doi:10.1016/j.yexmp.2018.03.006)
Supplement: Supplementary Table 1 — Features of libraries generated using Illumina TruSEQ or Rubicon Genomics Thruplex kits. ΔCt = difference between the cycle threshold of test to the control template ACD1 provided in the kit. ng = nanogram, PCR = polymerase chain reaction, bp = base pairs, nM = nanomoles. Supplementary Table 2 Comparing the DNA input requirement and cost for different commercial platforms available for copy number profiling. sWGS – shallow whole genome sequencing. Supplementary Table 3 Description of the libraries generated using the Rubicon Genomics Thruplex kit. Data given in range and median in brackets. ΔCt = difference between the cycle threshold of test to the control template ACD1 provided in the kit. ng = nanogram, bp = base pairs, nM = nanomoles. [file mmc7.docx]

Supplementary Table 1

| Kit | Numbers | DNA input (ng) | DNA Quality (ΔCt) | PCR cycles | Fragment Size (bp) | Library Yield (nM) |
| --- | --- | --- | --- | --- | --- | --- |
| Illumina TruSEQ | 45 | 50-500 | 0.4-6.7 | 8 | 293-391 | 0.0-56.9 |
| Rubicon Genomics Thruplex | 446 | 3.8-107 | -3.2 – 17.1 | 5-14 | 180-324 | 0.0-924.0 |

Supplementary Table 2

| Kits | Application | Required DNA | Cost array per sample (based on 100 samples) |
| --- | --- | --- | --- |
| Agilent 244k | Microarray | 200ng | £517 |
| Affymetrix Oncoscan FFPE | Microarray | 200ng | £460 |
| Nimblegen | Microarray | 1.5-2.5μg | Not available since 2013 |
| Illumina Infinium 850k | Microarray | 200ng | £128 |
| Illumina TruSEQ FFPE | sWGS | 50-500ng | £50 |
| Rubicon Genomics Thruplex DNAseq | sWGS | 50pg-50ng | £55 |

Supplementary Table 3

| Libraries | Numbers | DNA Input (ng) | DNA Quality (ΔCt) | Library Yield (nM) | Fragment Size (bp) |
| --- | --- | --- | --- | --- | --- |
| Very Good | 237 | 3.8-100 (30.3) | -0.3-12.7  (4.1) | 0-107.5 (8.5) | 180-288 (233) |
| Good | 142 | 4.3-107 (35.9) | 1-17.1  (4.2) | 0-77.2  (3.1) | 195-324 (229) |
| Intermediate | 47 | 4.0-59 (32.6) | -3.2-6.3  (3.5) | 0.1-924.8 (2.5) | 191-258 (231) |
| Poor | 12 | 14.7-50 (28.7) | 2-11.3  (4.9) | 0-13.0  (1.5) | 187-243 (219) |
| Failed | 8 | 4.8-51.1 (20.0) | 1.9-8.2  (4.3) | 0-24.2  (1.6) | 204-226 (221) |
